# Metabolomic evaluation of PGPR defence priming in wheat (*Triticum aestivum* L.) cultivars infected with *Puccinia striiformis* f. sp. *tritici* (stripe rust)

**DOI:** 10.3389/fpls.2023.1103413

**Published:** 2023-04-12

**Authors:** Manamele D. Mashabela, Fidele Tugizimana, Paul A. Steenkamp, Lizelle A. Piater, Ian A. Dubery, Tarekegn Terefe, Msizi I. Mhlongo

**Affiliations:** ^1^ Research Centre for Plant Metabolomics, Department of Biochemistry, University of Johannesburg, Johannesburg, South Africa; ^2^ International Research and Development Division, Omnia Group, Ltd., Johannesburg, South Africa; ^3^ Division of Small Grain Diseases and Crop Protection, Agricultural Research Council-Small Grains Institute (ARC-SGI), Private Bag X29 Bethlehem, Free State, Bethlehem, South Africa

**Keywords:** metabolic reprogramming, plant priming, plant growth promoting rhizobacteria, induced systematic resistance, ultra-high performance liquid chromatography-high-definition mass spectrometry, *Puccinia striiformis* f. sp. *tricti*

## Abstract

Plant-microbe interactions are a phenomenal display of symbiotic/parasitic relationships between living organisms. Plant growth-promoting rhizobacteria (PGPR) are some of the most widely investigated plant-beneficial microbes due to their capabilities in stimulating plant growth and development and conferring protection to plants against biotic and abiotic stresses. As such, PGPR-mediated plant priming/induced systemic resistance (ISR) has become a hot topic among researchers, particularly with prospects of applications in sustainable agriculture. The current study applies untargeted ultra-high performance liquid chromatography-high-definition mass spectrometry (UHPLC-HDMS) to investigate PGPR-based metabolic reconfigurations in the metabolome of primed wheat plants against *Puccinia striiformis* f. sp. *tricti* (*Pst*). A seed bio-priming approach was adopted, where seeds were coated with two PGPR strains namely *Bacillus subtilis* and *Paenibacillus alvei* (T22) and grown under controlled conditions in a glasshouse. The plants were infected with *Pst* one-week post-germination, followed by weekly harvesting of leaf material. Subsequent metabolite extraction was carried out for analysis on a UHPLC-HDMS system for data acquisition. The data was chemometrically processed to reveal the underlying trends and data structures as well as potential signatory biomarkers for priming against *Pst*. Results showed notable metabolic reprogramming in primary and secondary metabolism, where the amino acid and organic acid content of primed-control, primed-challenged and non-primed-challenged plants were differentially reprogrammed. Similar trends were observed from the secondary metabolism, in which primed plants (particularly primed-challenged) showed an up-regulation of phenolic compounds (flavonoids, hydroxycinnamic acids-HCAs- and HCA amides) compared to the non-primed plants. The metabolomics-based semi-quantitative and qualitative assessment of the plant metabolomes revealed a time-dependent metabolic reprogramming in primed-challenged and primed-unchallenged plants, indicating the metabolic adaptations of the plants to stripe rust infection over time.

## Introduction

Yellow stripe rust (SR) is an economically important disease affecting global wheat production and a potential threat to global food security. SR is caused by the obligate biotrophic fungus *Puccinia striiformis* f. sp. *tricti* (*Pst*), which has been reported in more than 64 countries from 1939 to 2016; in 2020, Zimbabwe became the latest addition to the list of affected countries (Boshoff et al., 2003; Carmona et al., 2020; Jamil et al., 2020). Over the years, the devastating impact of *Pst* on wheat production has left over 80% of existing wheat susceptible leading to annual losses (10% to 100%) of up to 5 million tons with an estimated cost of USD 1 billion (Beddow et al., 2015; Jamil et al., 2020). Epidemics of *Pst* are prevalent in spring and winter wheat; however, due to its obligate biotrophy, *Pst* also depends on summer and winter plants to complete its life cycle, which depends on two distinct plant hosts (Liu et al., 2015). Furthermore, *Pst*’s optimal growth requires regions with cool and moist temperate climates, at optimum temperatures of 12-20°C and relative humidity of 60% (Chen et al., 2014; Naseri and Kazemi, 2020). According to Schwessinger (2016), *Pst* completes its sexual life cycle on an alternative such as barberry (*Berberis* spp.) initiated by short-lived basidiospores and the subsequent development of aecium. The resulting aeciospores complete the asexual cycle by infecting the primary host, wheat cultivars (Chen et al., 2014; Rodriguez-Algaba et al., 2014).

The engineering of soil microbial communities has emerged as a viable eco-friendly and sustainable method for pathogen control. Interest in the use of PGPR in plant growth and development and as biocontrol agents (BCAs) has grown in recent years. The interaction of these plant-beneficial bacteria with both the plant and the invading pathogenic microbe generates a phenomenon known as plant-microbe tripartite interactions (plant–phytopathogen–beneficial microbe), which generally occur in the rhizosphere of the host plant (Mashabela et al., 2022a). On the other hand, plant-mediated PGPR interactions with non-soil-borne (aboveground) pathogens can occur in which a pathogenic infection on aboveground tissue of a host plant induces the exudation of relevant metabolites as stress signalling compounds to recruit suitable strains of PGPR for a defence response. Consequently, the beneficial microbes (PGPR) produce and secrete defence-related metabolites that induce a defence-oriented metabolic reprogramming in the host plant to mitigate the impact of the invading pathogen. This phenomenon is referred to as induced systemic resistance (ISR) through plant (bio) priming. In recent years, researchers have reported on the potential of PGPR as BCAs in agriculture; for instance, the effectiveness of *Bacillus cereus* as a BCA was demonstrated by Xu et al. (2014) against wheat sharp eyespot disease. The study reported reduced disease incidence in *B. cereus*-inoculated wheat plants by 31.4%, suggesting strong biocontrol effects by the *Bacillus* strain. Additionally, Mavrodi et al. (2012) evaluated PGPR antimicrobial activity against root rot pathogens of wheat. The study showed the ability of 11 *Pseudomonas* strains to reduce the disease symptoms and suppress *Rhizoctonia oryzae* and *Pythium irregularare*. Moreover, inhibition of both *R. solani* and *P. ultimum* pathogens was observed by reducing seedling damage, as evidenced by lowered disease rating score.

The current study aims to investigate the metabolic perturbations leading to plant priming and ISR in PGPR-treated wheat cultivars in response to *Pst* infection in an indirect tripartite plant-microbe interaction using untargeted metabolomics approaches. Seed-biopriming was carried out with two PGPR strains of *P. alvei* (T22) and *B. subtilis*, followed by infection with *Pst*. Metabolomics studies in this regard would unravel the metabolic pathways and biochemical mechanisms partaking in PGPR-induced ISR and plant priming. The study also explores a seed priming approach to PGPR treatment, a biopriming mechanism that involves pre-sowing treatment of seeds in biologically active bacterial inoculants in a priming solution (Akbar et al., 2019). Seed priming serves as a controlled mechanism of seed imbibition of a favourable formulation of compounds beneficial to improved seed germination, growth, yield parameters and, recently, systemic response to biotic and abiotic stress (Rakshit et al., 2015; Akbar et al., 2019).

## Materials and methods

### Wheat seed PGPR treatment

PGPR treatment of wheat seeds with strains *P. alvei* (T22) and *B. subtilis* was carried out as described by Mashabela et al. (2022b). Briefly, seeds were washed in sterile autoclaved water and then soaked in 50 mL of resuspended bacterial culture for biopriming. Coated seeds were dried at 28°C for 24h before planting. The selection of the two PGPR strains was based on previously published literature detailing their effectiveness as BCAs against a range of phytopathogens. The biocontrol capabilities of *P. alvei* have been demonstrated by Tugizimana et al. (2019) and Mhlongo et al. (2020b). Furthermore, *B. subtilis* has been reported on several occasions to significantly reduce yellow rust disease severity as a bio-fungicide (Reiss and Jørgensen, 2017; Omara et al., 2020).

### Plant growth and infection with *Puccinia striiformis*

*P. striiformis* spore were maintained in the ARC-SG rust collection, were multiplied on a susceptible wheat cultivar Avocet S. Fresh urediniospores collected from Avocet S were then inoculated onto the cultivars used in this study. The inoculated seedlings were placed in a cold chamber at ±10°C and about 100% relative humidity for 24 hours. Seedlings were then moved to a glasshouse cubicle and maintained in a day and night temperatures of ±18. For inoculation, dried spores were resuspended in Soltrol-170^®^ isoparaffininc carrier mineral oil (Chevron Phillips Chemical Company, Belgium) at a concentration of 5 mg spores/mL (6x10^6^ spores/mL). resuspended spores were pressure inoculated on wheat seedlings using a Vacuumbrand^®^ pressure pump, while control plants were mock-treated with only Soltrol-170^®^ mineral oil. The plants were then incubated in a dark cold room at 10-15°C overnight, then transferred to a glass house maintained at 15-18°C. PGPR-treated and control plants were grown in germination soil mixture in three biological replicates, and plant infection was carried out one-week post-germination. Infection was done one post germination, decause seedling resistance (all-stage resistance) can be expressed on seedlings at first leaf growth stage. So, inoculation was done seven days after planting (first leaf fully extended) (Boshoff et al., 2003). Lastly the samples were harvested every week for three consecutive weeks and store at -80°C until metabolite extraction.

### Sample preparation and data acquisition

Metabolite extraction and sample preparation were performed according to Mashabela et al. (2022b), followed by data acquisition where two microliters of 50% methanol extracts and QC samples were analysed on a Waters Acquity UHPLC system fitted with an ACQUITY UPLC HSS T3 column (1.8 µm x 2.2 mm x 150 mm) at a column temperature range of 20-60°C (Waters, Manchester, UK), hyphenated to a SYNAPT G1 quadrupole time-of-flight (Q-TOF) high-definition mass spectrometer (Waters Corporation Milford, MA, USA). The instrument parameters were as detailed by Mashabela et al. (2022b). The samples were analysed from the three biological replicates per sample, and each biological replicate analysed in three technical replicates such that n=9.

### Multivariate data analysis

A similar procedure for data pre-processing, mining, and application of MVDA as detailed in by Mashabela et al. (2022b) was adopted with minor modifications. In summary, pre-processing of UHPLC-Q-TOF-MS raw data was performed on MarkerLynxTM software (version 4.1, Waters Corporation, Milford, MA, USA). The following parameters were followed to generate the data matrices: Rt range of 0.68–26.35 min, a mass range of 100–1200 Da, a mass tolerance of 0.05 Da, and noise elimination level of 10 followed by data normalization on MassLynx XMTM software (Waters, Manchester, UK). Processed data matrices were exported for MVDA (chemometric analysis) on MetaboAnalyst, and PLS-DA modelling was performed for data dimension reduction (data visualization) and significant feature extraction (features contributing to observed discrimination between PGPR and pathogen treated and non-treated groups of data). Furthermore, PLS-DA score plots were used to visualize group separations based on the varying experimental conditions of the samples under investigation. The data was normalized, *Pareto*-scaled, and log-transformed before analysis. Lastly, metabolite annotation and compound identification were performed as outlined by Mashabela et al. (2022b).

## Results

### *Pst* infection of PGPR-primed plants and symptom development

The current study investigates the priming effects of PGPR on plants responding to biotic stress (*Pst*) challenge. Mashabela et al. (2022b) showed potential differential metabolic stress response mechanisms of susceptible plant cultivars compared to their resistant counterparts. These findings from metabolic analyses were further reflected by the visual inspection of the plant phenotypes, which showed that resistant cultivars develop chlorosis and necrosis at the site of infection to limit nutrient availability and thus reducing pathogen proliferation. For this study (to induce resistance in susceptible plant varieties), the *Pst*-susceptible Gariep cultivar was selected for further evaluation. Symptom development on PGPR-primed *Pst*-inoculated plants was monitored from 14 days post-infection (d.p.i). Visual inspection of PGPR-primed Gariep plants showed results that coincide with observations from the Koonap plants in the previous study; here, the primed plants developed leaf chlorosis and necrosis at the site of infection (**Figure S1A**) as well as a reduction in the infection rate evidenced by the development of smaller and fewer spores and reduced proliferation on the leaf surface compared to the non-primed Gariep plants (**Figure S1B**). The observed phenotypic traits of the PGPR-primed plants were thus a demonstration of metabolic changes associated with induced resistance against *Pst* in the primed plants.

### Metabolic profiling and chemometric analysis of PGPR-primed wheat plants responding to *Pst* infection

A study by Mashabela et al. (2022b) demonstrated the importance of metabolomics approaches in elucidating plant-microbe interactions and the subsequent plant (primary and secondary) metabolic perturbations in association with these interactions. The current study applies the analytical capabilities of untargeted metabolomics to elucidate the priming effect of PGPR on plants at a biochemical level. Methanolic extracts of *B. subtilis-* and *P. alvei (T22)*-primed Gariep plants challenged with *Pst* were chromatographically separated on an UHPLC system, followed by an MS detection in both ESI+ and ESI- ionisation modes (negative data presented). UHPLC-MS analysis generated base peak intensity (BPI) chromatograms (**Figure S2**), which revealed different chromatographic profiles between the PGPR-primed control, primed-infected (SRT or stripe rust treated) and non-primed-infected plants. The BPIs paint a picture of differential metabolite profiles based on the qualitative and quantitative variations in the accumulation of metabolites indicated by varying peak intensities and the presence or absence of certain metabolites. The observed chromatographic trends thus suggest plant priming related metabolic changes and metabolic alterations resulting from *Pst* infection.

**Figure 1:**
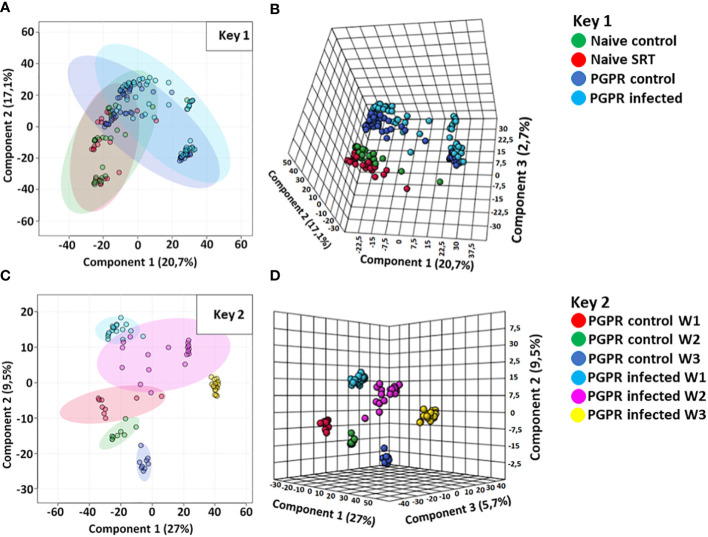
Computed principal component analysis (PCA) and partial least squares discriminant analysis (PLS-DA) models. **(A, B)** are computed PCA models showing the separated metabolic features of the non-primed (naive) control plants (red), non-primed infected/SRT plants (green), PGPR-primed control (dark blue) and PGPR-primed infected/SRT plants (light blue) from the *Pst* susceptible Gariep cultivar. **(C, D)** are 2D and 3D PLS-DA scores plots showing the time-dependent metabolic changes in PGPR-primed-challenged (infected) (light blue = week1; purple = week 2; yellow = week 3) compared to PGPR-primed-unchallenged (control) samples (red = week1; green = week 2; dark blue = week 3). Both sample groups show metabolic changes in the host plant over time, while indicating differences in the primed plants’ metabolic profiles compared to non-primed plants, as well as the differential metabolic reconfigurations occurring in plants based on pathogen infection. The data projected above were median-normalised, log transformed and Pareto-scaled in MetaboAnalyst for correlation and predictability scores of R^2^ = 0.6093 and Q^2^ = 0.5248 **(A)**; R^2^ = 0.8844 and Q^2^ = 0.7554 **(B)**; R^2^ = 0.7070 and Q^2^ = 0.5102 **(C)**; R^2^ = 0.8926 and Q^2^ = 0.6729 **(D)**.

The data were further analysed with chemometric models inclusive of a principal component analysis (PCA) and partial least squares-discriminant analysis (PLS-DA) methods. The chemometric modelling techniques enabled data dimensionality reduction, allowing the visual interpretation and further exploration of the multidimensional data into two- and three-dimensional representation (Madala et al., 2012), as computed in **Figure 1**. The generated models reveal underlying trends and patterns within the complex dataset, thus extracting essential information about the relationships (similarities and differences) between and within experimental sample groups. The two component PCA scores plot (**Figure 1A**) shows the differential clustering of similar and different samples from varying biological conditions (PGPR-primed-unchallenged, non-primed-unchallenged, PGPR-primed-challenged and non-primed-challenged) based on their distinct metabolic profiles. However, from the inspection of the 2D PCA scores plot, the first and second components also show overly saturated clustering of the data points from which clear differentiation between treated and untreated sample groups which makes it challenging to distinguish one sample group from another. As such, a 3D three component PCA scores plot was computed (**Figure 1B**), showing a clearer separation of the different sample groups. The observed separations further indicate metabolic perturbations in the PGPR-primed-unchallenged (control) and PGPR-primed-challenged (infected) plants, as well as in the PGPR-primed plants compared to their non-primed counterparts as previously suggested by the BPI chromatograms.

### Time-dependent metabolic reprogramming in primed-control vs. primed-infected wheat plants responding to *Pst* treatment

**Figure 2:**
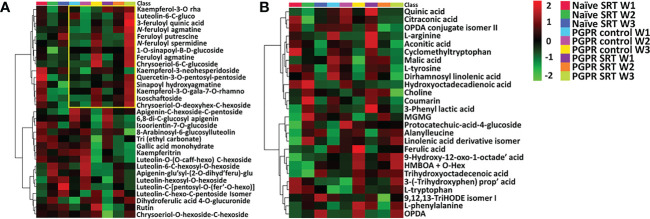
Interactive heatmap analysis of putatively annotated metabolite profiles from primed and non-primed infected and control Gariep cultivar samples. **(A)** A heatmap showing the differential qualitative and quantitative distribution of phenolic compounds and flavonoids in the PGPR-primed control and infected (SRT) plants vs. non-primed infected (naïve SRT) plants. **(B)** A heatmap that describes the relative quantification of annotated metabolites (amino acids, organic acids, fatty acids and benzenoid derivatives) detected in primed and non-primed Gariep samples over a three-week timepoint following *Pst* infection. Upregulated metabolites are shown in red whereas downregulated metabolites are indicated by a green rating on the heatmap, black shading shows no changes in the metabolite concentrations. The data projected above were median-normalised, log transformed and Pareto-scaled in MetaboAnalyst. The heatmap analysis thus showed the time-dependent perturbations in metabolic profiles of wheat plants due to plant priming and pathogen infection, compared to non-primed plant samples.

In addition to unravelling the variations in the metabolic profiles of plants based on their primary experimental conditions (treatments), the current study further aimed to investigate the time-course metabolic perturbations associated with plant priming and the subsequent defence response of PGPR-primed-challenged plants vs. PGPR-primed-unchallenged plants following *Pst* inoculation. As a point of reference, data from non-primed-challenged plants was included as a baseline for comparison. A time-course metabolic evaluation of the reprogrammed plant metabolome was carried out for samples harvested over a three-week period and attention was paid to the semi-quantitative distribution of annotated metabolites (**Table S1**) across the different experimental conditions and the respective time points. Interactive heatmaps (**Figure 2**) were generated in MetaboAnalyst 5.0 and showed differential accumulation of metabolites spanning both the primary (amino acids, organic acids and lipids-**Figure 2B**) and secondary (phenylpropanoids and flavonoids-**Figure 2A**).

Examination of the heatmaps revealed an increase in the concentrations of certain secondary metabolites in the PGPR-primed plants shown in yellow demarcation (**Figure 2A**). The levels of these specialised metabolites remained relatively unchanged in primed-unchallenged (control) plants in week 1 and 2, with a sharp increase by week 3. On the other hand, primed-challenged plants show an immediate, however be it gradual, increase in secondary metabolites from week 1 through to week 3, and a direct comparison shows higher overall content of these metabolites in the PGPR-primed-challenged plants relative to their unchallenged counterparts. Similarly, a study by Lephatsi et al. (2022) observed the upregulation of secondary metabolites such as flavonoid- and phenolic acids in plats treated with PGPR-based biostimulants. The accumulation of specialised metabolites thus indicates a prompt and continual defence response by the primed-challenged plants to *Pst* infection.

Time-dependant changes in the primary metabolism were also observed as an effect of PGPR-priming. TCA cycle intermediates such as citraconic acid, malic acid, aconitic acid and phenyllactic acid decreased over time, particularly in primed-challenged plants compared to unchallenged and non-primed plants. This trend, as seen in **Figure 2B** could be an indication of the redirection of the primary metabolism to feed the secondary metabolic pathways for the production of specialised defence metabolites, and this coincides with the increased accumulation of amino acids phenylalanine and tryptophan in the primed-challenged plants. Similar results were reported by Mhlongo et al. (2020a), where the inoculation of tomato plants with PGPR induced an increased accumulation of aromatic amino acids such as phenylalanine and tryptophan. Furthermore, lipid metabolism showed to have been impacted by plant priming; here there is an observed increase in fatty acids from the primed-challenged plants which assumed a gradual week to week accumulation compared to primed-unchallenged plants and a stark contrast between PGPR-primed-challenged compared to non-primed-challenged plants (**Figure 2B**). The observed differential regulation of metabolites thus correlates to the variations in metabolic profiles of the plants as previously alluded to, the time-course metabolic reconfiguration can also be observed in the PLS-DA models **Figures 1C, 1D** and BPI chromatograms in **Figures S2, S3** which show the class separations and time-variable differences in metabolic profiles between the sample groups over time, spanning both the primary and secondary metabolis.

**Figure 3:**
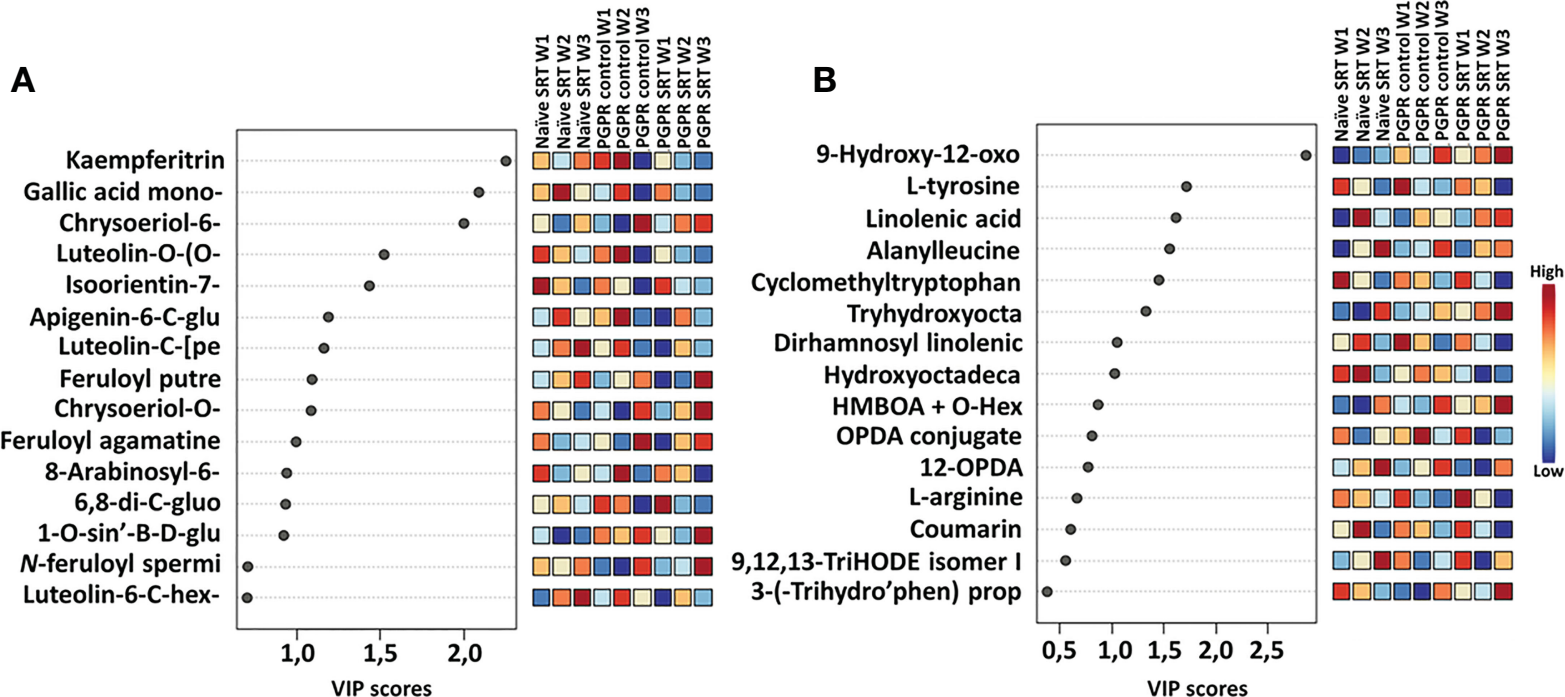
VIP score-plots of discriminant features derived from the PLS-DA analysis. The VIP scores display discriminant features in PGPR-primed-unchallenged (control) vs. PGPR-primed-challenged (SRT) plants vs. non-primed infected (SRT) plants for the Gariep cultivar samples over a period of three weeks post-infection. Selected metabolites (VIP score ≥ 1) in infected plants were compared to those in control plants at the given time point. The figures show differential accumulation of significant secondary **(A)** and primary **(B)** metabolites in infected plants. Some metabolites increased over time while others decreased. In addition to the effects of priming on the plant metabolome, *Pst* infection also induced the up- and down-regulation of metabolites in primed as compared to their non-primed counterparts. The data projected above were median-normalised, log transformed and Pareto-scaled in MetaboAnalyst.

The extraction of important (significant) metabolic features was made possible with VIP scores derived from PLS-DA analysis displaying discriminant features from the PGPR-primed control, PGPR-primed-challenged (SRT), PGPR-primed-unchallenged (control) and the non-primed infected (SRT) plant samples (**Figure 3**). The VIP scores are useful in selecting the most substantially changed (decreased or increased) metabolites leading to the observed differences in the metabolic profiles of samples. Selected metabolites with VIP scores of >1 also present potential significant biomarkers for the plant response and priming effect of PGPGR against pathogen infection.

**Figure 4:**
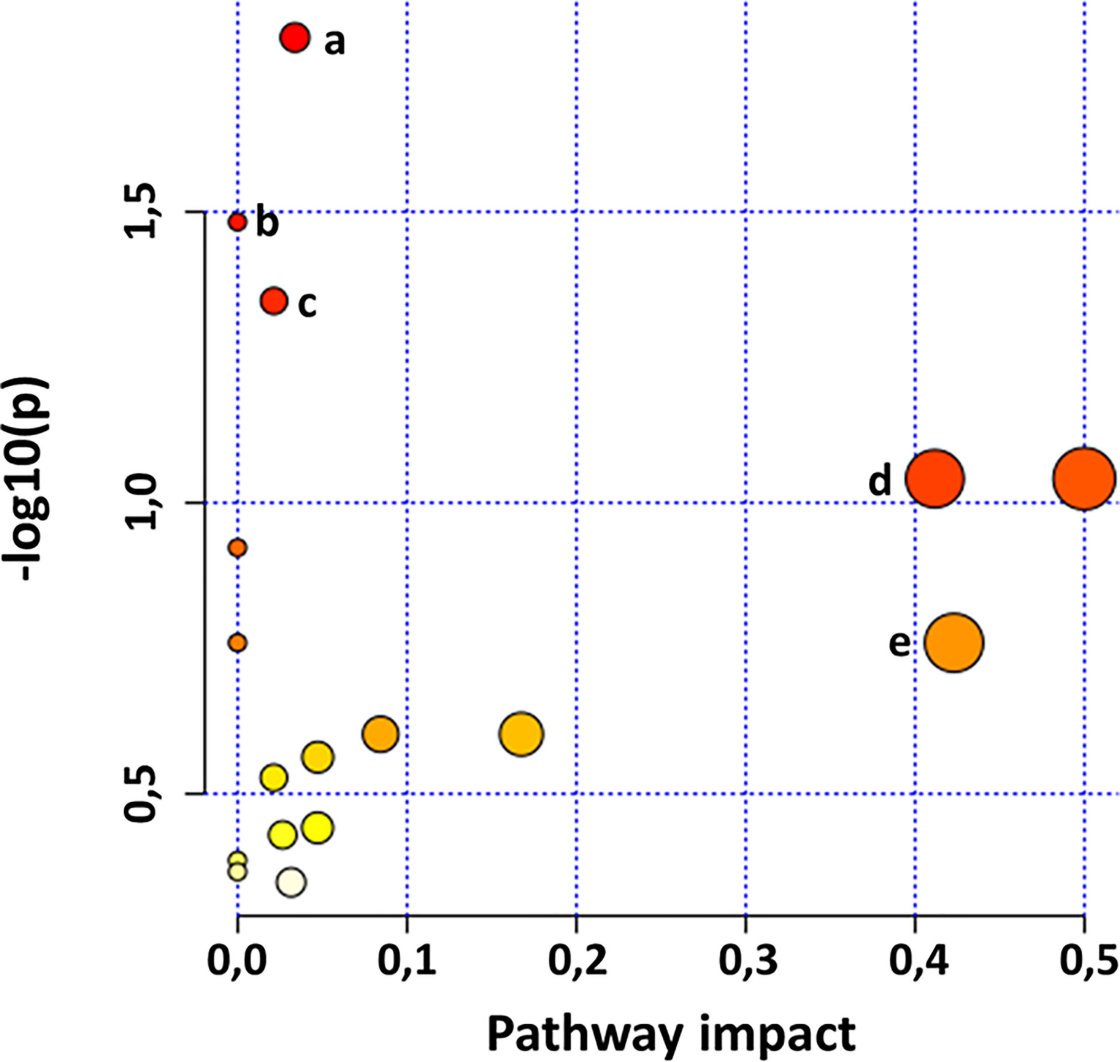
MetaboAnalyst-computed pathway analysis. View of statistically significant pathways flagged from the metabolome view based on matched metabolites. The figures show pathways matched from the annotated Gariep metabolites. The pathways are arranged based on the p-value (y-axis), which indicates the pathway enrichment analysis, and pathway impact values (x-axis) representing pathway topology analysis. The node colour of each pathway is determined by the p-value (red = lowest p-value and highest statistical significance), and the node radius (size) is based on the pathway impact factor, with the biggest indicating the highest impact. Some of the most significantly impacted pathways are **(A)** phenylpropanoid biosynthesis, **(B)** aminoacyl-tRNA biosynthesis, **(C)** phenylalanine, tyrosine and tryptophan biosynthesis, **(D)** isoquinoline alkaloid biosynthesis and **(E)** phenylalanine metabolites.

Chemometric analysis has thus far assisted in discriminating metabolic features and profiles of plant samples, showing the impact of PGPR-based plant priming on the changes in the general metabolome of said plants over time and the dynamics of metabolic reprogramming for defence response in primed plant compared to their non-primed counterparts. As such, an evaluation of the impact of the phenomenon previously mentioned on the metabolic pathways of plants was required to investigate the pathways impacted by these experimental conditions that lead to the observed metabolic perturbations. Metabolic pathway analysis (MetPA) was performed in MetaboAnalyst using matched HMDB IDs of putatively annotated metabolites (**Table S1**) for the identification and visualisation of the most significantly impacted pathways (p-value < 0.05) as reflected by the apparent metabolic reprogramming. The resulting impacted metabolic pathways (**Figure 4**) show the important role of certain metabolites such as phenylalanine and tyrosine in plant metabolism. These amino acids were found to be the pivotal metabolites impacting the plant metabolism through the phenylalanine, tyrosine and tryptophan biosynthesis pathway and the phenylpropanoid biosynthesis pathway. Additionally, phenylalanine and tyrosine serve as a links between these two pathways connecting the primary and secondary metabolism. As such, an impact on the biosynthesis of these aromatic amino acids, be it qualitative or quantitative, can effect metabolic changes across the metabolome of the host plant. For instance, tyrosine was found to be a significant discriminant feature (**Figure 3B**) and was found in higher quantities in non-primed plants compared to PGPR-primed-unchallenged and PGPR-primed-challenged plants. Since the impacted primary metabolism pathway feeds into the impacted secondary metabolism pathway, this indicates that the plant priming induced a redirection of metabolic activity towards the production of defence metabolites as also indicated by the increased accumulation of phenolic compounds in the primed plants over time (**Figure 2A**).

## Discussion

The findings by Mashabela et al. (2022b) gave insights into the potential response mechanisms of *Pst*-resistant and susceptible wheat cultivars to pathogen infection through morphological (phenotypic) and biochemical display, or lack thereof of defence strategies. These defence mechanisms are inclusive of HR-mediated responses which induce rapid cell death at the site of infection as a means of defence against pathogens. The study reported the lack, or delayed deployment of HR in *Pst*-susceptible cultivars to be a favourable contributing factor to pathogen invasion and proliferation as aided by sufficient nutrient availability for spore germination and development. Therefore, successful expression of HR in resistant varieties at both the biochemical and phenotypic levels could serve as a mechanism for resistance against *Pst* not available in the susceptible Gariep cultivars.

As such, the current study sought to apply the previously reported plant protective capabilities of PGPR through induced systemic resistance (ISR) and plant priming (Mashabela et al., 2022a), to induce a heightened defence response in Pst-susceptible wheat cultivars, and to elucidate the resulting metabolic perturbations occurring due to PGPR-based plant priming. Metabolic reprogramming is required for plant adaptation to environmental conditions, such perturbations are generally induced through the perception of an external stimulus, the case for PGPR in ISR, which triggers the differential regulation of metabolites and metabolic pathways based on the current (nutritional or defence) requirements (Mashabela et al., 2022a).

### Time-dependant differential metabolic reprogramming of PGPR-primed vs. non-primed plants challenged with *Pst* compared to primed controls plants

An untargeted LC-MS analysis of Gariep plant samples aided in the putative annotation of metabolites from the primary metabolism. Annotated metabolites were differentially distributed among the samples based on the varying experimental conditions. For instance, the aromatic amino acids phenylalanine and tyrosine were annotated from the three sample groups of plants (PGPR-primed-unchallenged, PGPR-primed-challenged, and non-primed-challenged). Evaluation of the amino acid distribution revealed differential time-dependant accumulation of the compounds, with an observed up-regulation of phenylalanine in PGPR-primed-challenged and PGPR-primed-unchallenged plants compared to non-primed-challenged plants. The increased levels of phenylalanine could serve as a pivotal role in plant defence signalling for induced resistance, based on its function as the precursor for the phenylpropanoid biosynthesis pathway (López-Gresa et al., 2010). Elevated levels of this amino acid thus avail more substrate material for a rapid and sustained biosynthesis of specialised secondary defence metabolites such flavonoids, HCA derivatives and other phenolic compounds (Tugizimana et al., 2019; Mhlongo et al., 2020a).

Interestingly, not only were the levels of phenylalanine lower in non-primed-challenged in contrast to PGPR-primed-challenged and PGPR-primed-unchallenged; a time-course quantitative assessment revealed the aromatic amino acid decreased over time. Depletion of this essential compound could result in lowered defence capacity due to reduced rates of defence metabolite biosynthesis in the non-primed plants. Moreover, both phenylalanine and tyrosine were higher in PGPR-primed-challenged compared to PGPR-primed-unchallenged. This observation further suggests that the primed-challenged plants produced more aromatic amino acids than primed-unchallenged plants to channel the biosynthesis of defence metabolites for induced resistance against *Pst*. On the other hand, tyrosine concentrations were higher in non-primed-challenged and relatively lower to a similar scale for PGPR-primed-challenged and PGPR-primed-unchallenged. A correlation heatmap analysis (not presented) showed a negative correlation between tyrosine and phenylalanine. The two amino acids share a direct precursor for biosynthesis in L-arogenate (C00826) in **Figure 4A**, which suggests that increased rates of phenylalanine biosynthesis could lead to reduced levels of tyrosine and *vice versa*. Essentially, primed plants favoured the production of phenylalanine, thus possibly accelerating the subsequent biosynthesis of secondary metabolites such as phenylpropanoids for defence response and resistance against *Pst*. According to Simpson et al. (2021), both phenylalanine and tyrosine are precursors of similar secondary metabolites. However, phenylalanine is favoured to produce the most common secondary metabolites in plants, such as HCAs and flavonoid glycosides. additionally, phenylalanine is particularly significant due to its ability to switch from the primary to the secondary metabolism of the plant, enabling the production of a wide range of secondary metabolites (Feduraev et al., 2020).

L-arginine and L-tryptophan and tryptophan were also annotated in this study, and both amino acids have been reported to play pivotal roles in plant defence signalling and the biosynthesis of specialised secondary metabolites (Forde and Lea, 2007; Tzin and Galili, 2010; Parthasarathy et al., 2018; Mhlongo et al., 2021). Tryptophan metabolism results in the production of auxins, which are essential defence signalling molecules, as well as phytoalexins which are readily available in plants as pre-existing defence metabolites forming a defence barrier prior to pathogenic invasion (Ly et al., 2008; Tzin and Galili, 2010). As such, the reduced levels of tryptophan from primed plants indicate a continuous utilisation of the molecule to produce the required defence signalling molecules and phytoalexins for a more robust and quick defence response as compared to non-primed plants. Arginine was found to decrease over time in the primed plants, also indicating high utilisation rates of this amino acids. This coincides with the notable increase in polyamine derivatives (**Figure 3A**) observed in the primed plants. Arginine is metabolised into polyamines which are involved in plant defence and signalling against pathogens (Zeiss et al., 2021).

**Figure 5:**
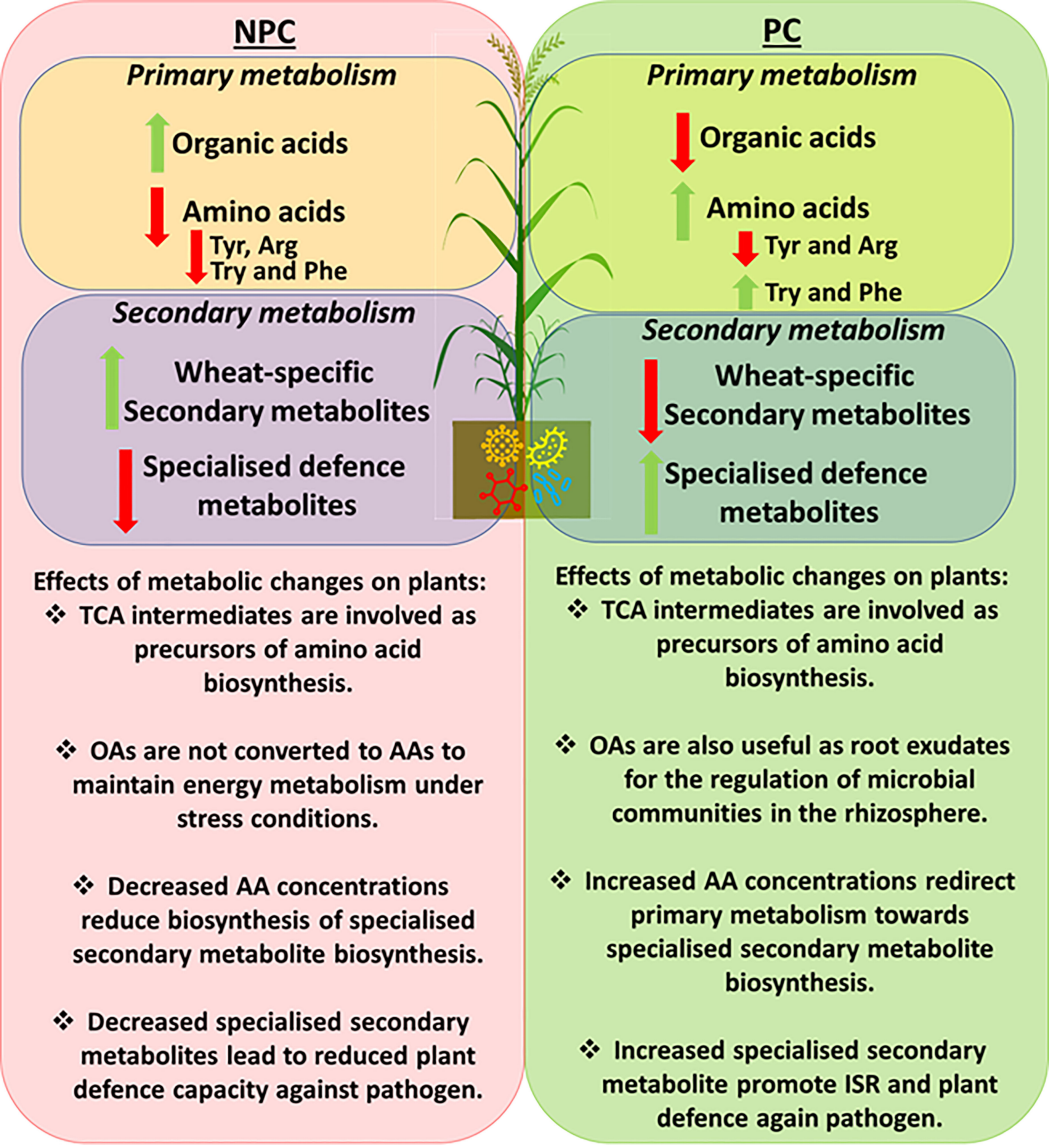
Summary diagram of PGPR-induced metabolic changes in plants responding to *Pst* infection. The left side highlights metabolic changes in the non-prime-challenged (NPC) plants responding to *Pst* infection. Increased levels of TCA organic acids (OAs) intermediates (malic acid, aconitic acid and citraconic acid) were observed, indicating a low conversion rate to amino acids as an indication of a conserved energy metabolism for plant growth. Ultimately, low OA conversion rate led to reduce amino acids (AAs) concentrations and the subsequent reductions in specialised secondary defence metabolites. An opposite effect was observed in primed challenged (PC) plants in which PGPR priming induced the accumulation of specialised secondary defence metabolites. This phenomenon may have occurred due to the rapid conversion of precursor amino acids towards secondary metabolites biosynthesis and thus induced systemic resistance against the invading pathogen.Conclusions.

Studies have reported that amino acids can serve as precursors for the biosynthesis of TCA intermediates (organic acids) which modulate mitochondrial metabolism and ATP production in plant cells (Hildebrandt et al., 2015; Killiny and Hijaz, 2016; Macias-Benitez et al., 2020; Yang et al., 2020). A metabolic interplay between amino acids and TCA intermediates such as malic acid, citraconic acid and aconitic acid annotated in this study, drives the central pathways for energy metabolism through carbohydrates catabolism and fatty acid oxidation, which are essential for maintaining the photosynthetic energy production capacity of plants (Zeiss et al., 2018; Ludwig et al., 2016; Nephali et al., 2021). Malic acid concentrations decreased in the leaves of PGPR-primed plants, while an increase was observed in the non-primed plants. According to Rudrappa et al. (2008), root-secreted malic acid recruits beneficial soil bacteria into the rhizosphere. Additionally, Sasse et al. (2018) reported that the increased malic acid exudation led to the attraction and successful root colonisation of the biocontrol agent *Bacillus subtilis*. Based on these reports, it can be speculated that decreased leaf malate content of PGPR-primed plants was due to malate exudation into the rhizosphere for chemotaxis properties and the maintenance of plant-PGPR symbiosis. Aconitic acid also provides various biological activities in plants as an antifungal and antifeedant (Bruni and Klasson, 2022). Moreover, metabolic reprogramming can redirect TCA cycle intermediates for aromatic amino acid biosynthesis geared towards the phenylpropanoid pathway to produce defence metabolites, or the production of fatty acids for lipid-mediated signal transduction and membrane disruption for HR (Mashabela et al., 2022b). These observations further suggest that PGPR plant priming induces metabolic reconfigurations in host plants for metabolite reallocation to aid in plant growth and development, but particularly for enhanced defence response mechanisms through ISR (**Figure 5**).

### Differential reprogramming of the secondary metabolism in PGPR-primed vs. non-primed plants challenged with *Pst* compared to primed controls plants

Changes in the secondary metabolism of PGPR-primed control, primed SRT and non-primed plants were observed. A combined untargeted LC-MS and chemometric analysis approach revealed time-dependant and treatment-based metabolic reprogramming led by the differential regulation of flavonoids, flavonoid glycosides, HCAs and polyamine derivative conjugates (HCAAs). The time-dependant metabolic reconfigurations are an indication of plant adaptation to the immediate environment, which facilitates a fine-tuned defence response against pathogenic infections (Mhlongo et al., 2021), through the plant’s developmental stages. Flavonoids and related flavones or isoflavones are highly constitutive specialised plant secondary metabolites with an arsenal of biological activities, particularly in plant defence response, including but not limited to, defence signalling, antioxidant, and antimicrobial activity, as well as biotic resistance and abiotic tolerance (Mashabela et al., 2022b). Increased levels of these phenolic compounds were observed in the primed plants over time, with higher concentrations in week 3 of the primed-challenged samples, while many of these compounds remained relatively unchanged in the non-primed plants. This indicates enhanced secondary metabolite production due to PGPR priming as well as in response to pathogen inoculation.

A time-course accrual of HCA derivatives was only apparent in the primed plants (**Figure 3A**). The defence capabilities of HCAs have been previously described (Zeiss et al., 2019) and have also been reported to drive priming mechanisms in plants (Mhlongo et al., 2016; Mhlongo et al., 2021). Most HCAs acquire functional properties in their allied forms; these compounds, also annotated in this study, are generally conjugated to sugars (1-O-sinapoyl-B-D-glucoside, dihydroferulic acid-4-O-glucuronide), polyamines (sinapoyl hydroxy agmatine) and quinic acids (feruloyl quinic acid) and are functional antimicrobial compounds as well as also involved as substrates in the biosynthesis of defence metabolites (Mhlongo et al., 2021). An accumulation of these compounds could lead to an enhanced defence response and thus induced resistance in the primed-SRT plants, this is based on their antioxidant and reducing properties which effect antimicrobial activity in the host plant (Groenbaek et al., 2019; Yang et al., 2020). Moreover, HCAs play a vital role in the formation of cross linkages in secondary cell walls as precursors of monolignols in lignin (Nigro et al., 2020; Mashabela et al., 2022b). The potential role of HCAAs or HCA amides in plant defence was reported by Mashabela et al. (2022b) following increased concentrations of these compound in the resistant Koonap cultivar compared to the susceptible Gariep cultivar. HCAAs such as feruloyl agmatine, feruloyl putrescine and sinapoyl hydroxy agmatine were annotated, these compounds have been reported to display antimicrobial activity and participate in cell wall strengthening (Facchini et al., 2002; Leonard et al., 2022). The accumulation of these compound in the primed-SRT-challenged plants could point to the priming effect of PGPR and induced defence response, thus conferring resistance against *Pst*, and serve as biomarkers for plant resistance against biotic stress (**Figure 5**).

Plant priming is an adaptive strategy that enhances the defence capacity of plants through the induction of an enhanced defence mechanism, and this phenomenon regulates the metabolome of plants which subsequently leads to a rapid and more robust defence response at the metabolic and physiological state of the primed plant (Aranega-Bou et al., 2014; Mauch-Mani et al., 2017). The priming effectors are generally chemical elicitors applied exogenously, however in recent years natural elicitors such as plant-beneficial microbes (including PGPR) have been investigated. Nevertheless, the mechanistic nature of the priming effects at the biochemical level are still largely elusive. The current study thus aimed to elucidate the chemical mechanisms underlying PGPR-mediated plant priming and the apparent metabolic reprogramming leading to induced systemic resistance. An untargeted metabolomics approach was used to analyse samples of PGPR-primed-control, primed-SRT and non-primed-SRT plants, revealing time-dependant metabolic reconfigurations in said plants. The changes spanned both the primary (amino acid, organic acid and fatty acids) and secondary (phenylpropanoids, flavonoids, HCAs and HCAAs) metabolism, resulting in priming- and pathogen infection-induced differential regulation of metabolites geared towards enhanced plant defence. Upon perceiving a priming stimulus, the plant undergoes metabolic reprogramming, which activates a cascade of defence signalling leading to a primed state that confers a robust and rapid response in plants upon challenge by a pathogen. The identified metabolic features and biomarkers could provide insights into the potential use of microbe-derived compounds for metabolic engineering to promote plant priming for sustainable agriculture.

## Data availability statement

The original contributions presented in the study are included in the article/**Supplementary Material**. Further inquiries can be directed to the corresponding author.

## Author contributions

Conceptualization: MDM and MIM, Methodology: MDM, PS, TT and MIM., Investigation: MDM, PS and MIM., Visualization: MDM., Funding acquisition: MIM., Project administration: MIM. Supervision: MIM., FT, I.A.D and LP, Writing – original draft: MDM, Writing – review & editing: MIM, FT, ID and LP. The authors read and approved the final manuscript. All authors contributed to the article and approved the submitted version.
