# Supplementary material for: Metabolomic evaluation of PGPR defence priming in wheat (Triticum aestivum L.) cultivars infected with Puccinia striiformis f. sp. tritici (stripe rust)
Source: Front Plant Sci. 2023 Apr 12;14:1103413. doi: 10.3389/fpls.2023.1103413 (PMC10132142; doi:10.3389/fpls.2023.1103413)
Supplement: Supplementary file 1 [file DataSheet_1.docx]

**Metabolomic evaluation of PGPR defence priming in wheat (*Triticum aestivum* L.) cultivars infected with *Puccinia striiformis* f. sp. *tritici* (Stripe rust)**

**Manamele D. Mashabela^1^, Fidele Tugizimana^1, 2^, Paul A. Steenkamp^1^, Lizelle A. Piater^1^, Ian Dubery^1^, Tarekegn Terefe^3^ and Msizi I. Mhlongo^1*^**

^1^Research Centre for Plant Metabolomics, Department of Biochemistry, University of Johannesburg, P.O. Box 524, Auckland Park, Johannesburg 2006, South Africa

^2^International Research and Development Division, Omnia Group, Ltd., Johannesburg 2006, South Africa

^3^Division of Small Grain Diseases and Crop Protection, Agricultural Research Council-Small Grains Institute (ARC-SGI), Private Bag X29 Bethlehem, Free State 9700, South Africa

***Correspondence:**Msizi Mhlongo, [mmhlongo@uj.ac.za](mailto:mmhlongo@uj.ac.za)

**Frontiers Supplementary file.**


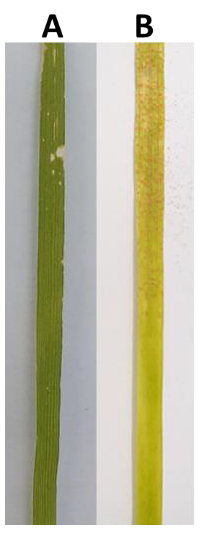


**Figure S1: Symptom development in primed and non-primed Gariep wheat cultivars inoculated with *Pst*.** Symptom development was monitored from 14 days post-infection. The PGPR-primed Gariep plants **(A)**, showed chlorosis and necrosis at the site of infection compared to the non-primed Gariep variety **(B)**. There was also a restricted development of *Pst* spores in both size and proliferation on the primed plants compared to their non-primed counterpart. The observations made from the symptoms suggests the induction of an HR in primed plants as a means of defence response against pathogenic infection.


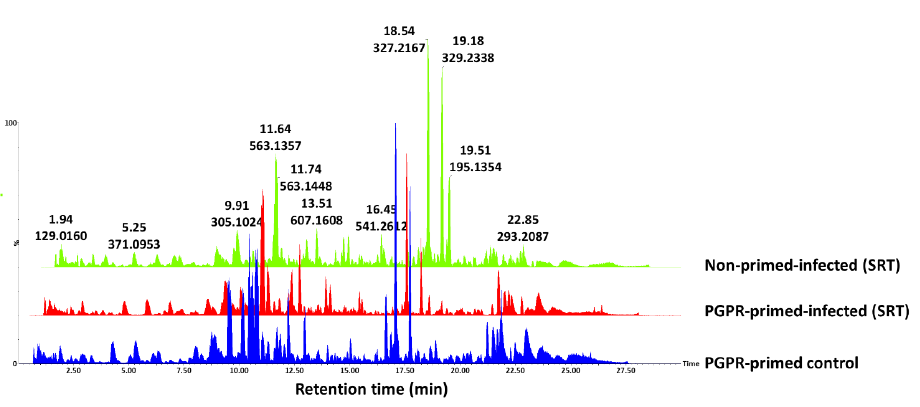


**Figure S2: ESI negative BPI MS chromatograms of PGPR-primed-control, PGPR-primed-infected *vs*. non-primed-infected plants.** The chromatograms show variations in the metabolic profiles of methanol extracts from PGPR-primed-control, PGPR-primed-infected vs. non-primed-infected Gariep cultivar plants.

**
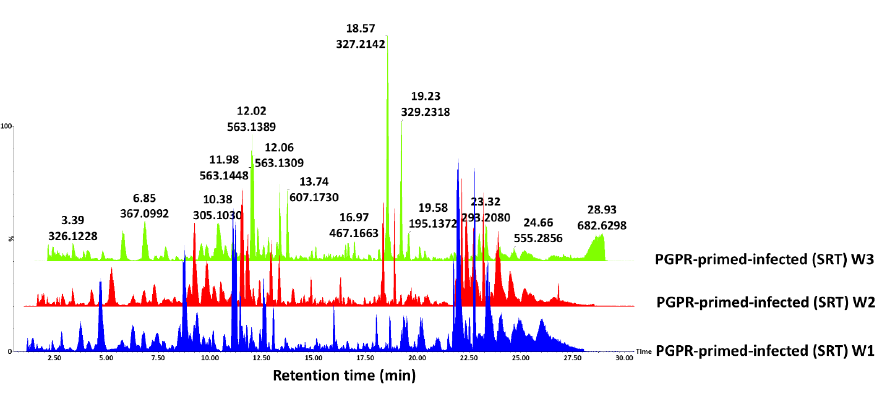
**

**Figure S3: ESI negative BPI MS chromatograms PGPR-primed-infected.** The chromatograms show time-dependent variations in the metabolic profiles of methanol extracts from PGPR-primed-infected Gariep cultivar over a three-week period.

**Table S1: Summary of the annotated, putatively identified metabolites (MSI-L2)**.

| **No.** | **Compound** | **Chemical Formula** | **Rt (min)** | ***m/z*** | **Adduct** | **Fragments (*m/z*)** |
| --- | --- | --- | --- | --- | --- | --- |
| **1** | L-Arginine | C6H15N4O2 | 0.75 | 175.1025 | [M+H]+ | 158, 130, 118, 116 |
| **2** | Choline | C5H14NO | 0.86 | 104.1062 | [M+H]+ | 60, |
| **3** | Adenine | C5H5N5 | 0.92 | 136.0658 | [M+H]+ | 118, |
| **4** | Citraconic acid | C5H6O4 | 0.98 | 129.0194 | [M-H]- | 85 |
| **5** | Malic acid | C4H6O5 | 1.04 | 133.0127 | [M-H]- | 191, 115 |
| **6** | Quinic acid | C7H12O6 | 1.24 | 191.0182 | [M-H]- | 173, 111 |
| **7** | Aconitic acid | C6H6O6 | 1.28 | 173.0889 | [M-H]- | 129, 117, 85 |
| **8** | Indoline | C8H9N | 1.85 | 120.0814 | [M+H]+ | 103, |
| **9** | L-Phenylalanine | C9H11NO2 | 2.03 | 164.0706 | [M-H]- | 147, 129, 103 |
| **10** | Protocatechuic acid-4-glucoside | C13H16O9 | 2.18 | 315.0755 | [M-H]- | 153, 108 |
| **11** | Pantothenic acid | C9H17NO5 | 2.23 | 220.1169 | [M+H]+ | 185, 116, 90 |
| **12** | Alanylleucine | C9H18N2O3 | 2.54 | 203.1404 | [M+H]+ | 157, 132, 86 |
| **13** | Indole-3-carboxaldehyde | C9H7NO | 2.98 | 146.0616 | [M+H]+ | 118, 91 |
| **14** | 3-(3,4,5-Trihydroxyphenyl) propanoic acid | C9H10O5 | 3.2 | 197.0412 | [M-H]- | 153, 151, 138, 123, 109 |
| **15** | L-Valine | C5H11NO2 | 3.35 | 118.0868 | [M+H]+ | 70, 55 |
| **16** | L-Tryptophan | C11H12N2O2 | 3.37 | 205.0968 | [M+H]+ | 188, 146, 118 |
| **17** | L-Leucine | C6H13NO2 | 3.87 | 132.0865 | [M+H]+ | 86, 69 |
| **18** | L-Glutamic acid | C5H9NO4 | 4.19 | 148.0580 | [M+H]+ | 130, 84, 72 |
| **19** | Coumarin | C9H6O2 | 4.19 | 147.0470 | [M+H]+ | 103, 91 |
| **20** | L-Tyrosine | C9H11NO3 | 4.24 | 182.0807 | [M+H]+ | 165, 136, 147, 123, 119, 91 |
| **21** | Feruloyl agmatine | C15H22N4O3 | 4.71 | 307.1698 | [M+H]+ | 307, 273, 177, 145 |
| **22** | Ferulic acid | C10H9O3 | 4.82 | 177.0552 | [M+H]+ | 145, 117, 89 |
| **23** | Cyclomethyltryptophan | C12H12N2O2 | 5.08 | 217.0977 | [M+H]+ | 144, |
| **24** | Feruloyl putrescine | C14H20N2O3 | 5.19 | 265.1552 | [M+H]+ | 248, 177, 145 |
| **25** | Tri (ethyl carbonate) | C16H18O11 | 5.37 | 385.0782 | [M-H]- | 297, 89 |
| **26** | *N-*Feruloyl spermaidine | C17H28N3O3 | 5.77 | 322.1654 | [M+H]+ | 321, 177, 163, 146, 117, 89 |
| **27** | 3-Indole acrylic acid | C11H9NO2 | 5.8 | 188.0712 | [M+H]+ | 146, 144, 118, 102, 72 |
| **28** | 3-Feruloyl quinic acid | C17H20O9 | 5.91 | 367.0970 | [M-H]- | 351, 219, 193, 178, 134, 102 |
| **29** | 3-Feruloyl quinic acid isomer | C17H20O9 | 5.92 | 367.1045 | [M-H]- | 193, 134 |
| **30** | Coumaroyl agmatine | C14H20N4O2 | 6.24 | 277.1623 | [M+H]+ | 260, 218, 147, 145, 131, 114 |
| **31** | 2-O-Glucosyl-7-methoxy-1,4(2H)-benzoxazin-3-one (HMBOA + O-Hex) | C15H19NO9 | 7.17 | 356.0978 | [M-H]- | 300, 194, 166, 138 |
| **32** | Saccharide compound | C16H20O10 | 7.29 | 371.0976 | [M-H]- | 249, 231, 121, 113 |
| **33** | Nicoblumin | C25H42O13 | 7.31 | 549.2547 | [M-H]- | 387, 227 |
| **34** | *N-*Feruloyl agmatine | C15H22N4O3 | 8.05 | 307.1725 | [M+H]+ | 290, 248, 247, 178, 177, 145, 117, 114, 95 |
| **35** | *N*-Acetyl-aspartyl glutamic acid | C11H16N2O8 | 8.06 | 303.0820 | [M-H]- | 303, 96 |
| **36** | 4-acetyl-2(3H)-Benzoxazolone (ABOA) | C9H7NO3 | 8.11 | 178.0497 | [M+H]+ | 150, 122, 95, 86 |
| **37** | Sinapoyl hydroxyagmatine | C16H24N4O5 | 8.59 | 351.1268 | [M-H]- | 249, 101 |
| **38** | Luteolin-6-C-hexoside-O-hexoside | C27H30O16 | 8.88 | 611.1612 | [M+H]+ | 449, 451, 413, 329 |
| **39** | Dihydroferulic acid 4-O-glucuronide | C16H20O10 | 9.25 | 371.0978 | [M-H]- | 195, 175 |
| **40** | Luteolin-C-hexoside-C-pentoside Isomer | C26H28O15 | 9.61 | 579.1350 | [M-H]- | 489, 459, 399, 369, 339 |
| **41** | 1-O-Sinapoyl-β-D-glucose | C17H22O10 | 9.81 | 385.1135 | [M-H]- | 223, 164 |
| **42** | 8-Arabinosyl-6-glucosylluteolin | C26H28O15 | 9.96 | 579.1332 | [M-H]- | 561, 489, 459, 399 |
| **43** | Kaempferol-3-O-galactoside-7-O-rhamnoside | C27H30O15 | 10.11 | 593.1525 | [M-H]- | 447, 283 |
| **44** | Luteolin-6-C-hexosyl-O-hexoside | C27H30O16 | 10.15 | 611.1700 | [M+H]+ | 593, 575, 545, 461, 431, 413, 395, 383, 353, 329, 299 |
| **45** | Kaempferol-3-O-rutinoside | C27H30O15 | 10.45 | 593.1498 | [M-H]- | 447, 300, 285, 284 |
| **46** | Isovitexin-7-O-glucoside | C27H30O15 | 10.45 | 593.1506 | [M-H]- | 473, 431, 341, 311, |
| **47** | Quercetin-3-O-pentosyl-pentoside | C25H26O15 | 10.46 | 565.1477 | [M-H]- | 447, 309, 285 |
| **48** | Hordatine-C-hexose isomer I | C44H33O16 | 10.52 | 771.2019 | [M-H]- | 771, 609, 593, 503, 473 |
| **49** | Rutin | C27H30O16 | 10.54 | 609.1469 | [M-H]- | 593, 447, 309, 285 |
| **50** | Apigenin C-hexoside-C-pentoside | C26H28014 | 10.6 | 565.1557 | [M+H]+ | 547, 529, 511 |
| **51** | Schaftoside | C26H27014 | 10.61 | 565.1670 | [M+H]+ | 427, 409,379, 337, 325 |
| **52** | Sinapoyl aldehyde | C11H12O4 | 10.84 | 209.0797 | [M+H]+ | 181, 177, 121 |
| **53** | Isoschaftoside | C26H28O14 | 10.86 | 563.1401 | [M-H]- | 473; 353; 325 |
| **54** | Luteolin-C-hexoside-O-deoxyhexoside | C27H30O15 | 10.86 | 595.1663 | [M+H]+ | 449, 431, 383, 353, 329, 299 |
| **55** | Luteolin-6-C-glucoside | C21H20O11 | 10.86 | 447.0917 | [M+H]+ | 431, 413, 353, 329, 299 |
| **56** | Kaempferol-3-neohesperidoside | C27H30O15 | 10.87 | 595.1775 | [M+H]+ | 449, 299, 229, |
| **57** | Iso-orientin | C21H20O11 | 11.16 | 447.0927 | [M-H]- | 429, 357, 327, 285 |
| **58** | Apigenin-6-C-glucosyl-8-C-(2''-O-dihydroferuloyl)-glucoside | C34H28O21 | 11.37 | 771.2049 | [M-H]- | 593, 503, 473, 383 |
| **59** | Kaempferol-3-O-glucoside | C21H20O11 | 11.4 | 447.0907 | [M-H]- | 285, 284, 255, 227 |
| **60** | 4-Coumaric acid | C9H8O3 | 11.44 | 165.0531 | [M+H]+ | 145, 123, 119, 103, 89, 69 |
| **61** | Loliolide | C11H16O3 | 11.58 | 197.1178 | [M+H]+ | 179, 161, 133, 107 |
| **62** | 6,8-di-C-glucosyl apigenin | C27H30O15 | 11.62 | 593.1506 | [M-H]- | 575, 473, 372 |
| **63** | Chrysoeriol-O-hexoside-C-hexoside | C28H32016 | 11.78 | 625.1769 | [M+H]+ | 463, 445, 427, 409, 397, 367, 343, 313 |
| **64** | Apigenin-6-C-glucoside | C21H20O10 | 11.84 | 433.1135 | [M+H]+ | 415, 397, 379, 349, 337, 313, 283 |
| **65** | Kaempferol-3-O-rhamnoside-7-O-rhamnoside | C27H30O14 | 11.91 | 577.1519 | [M-H]- | 431 |
| **66** | Vitexin-2''-O-rhamnoside | C27H30O14 | 11.93 | 579.1659 | [M+H]+ | 433, 415, 397, 367, 313, 204 |
| **67** | Isovitexin | C21H20O10 | 11.93 | 433.1105 | [M+H]+ | 415, 397, 367, 313, 204 |
| **68** | Kaempferitrin | C27H30O14 | 12.13 | 577.1557 | [M-H]- | 563, 453, 431, 413, 355, 341, 293, 283 |
| **69** | Chrysoeriol-O-deoxyhexoside-C-hexoside | C28H32O15 | 12.28 | 609.1819 | [M+H]+ | 463, 445, 427, 409, 397, 367, 343, 313 |
| **70** | Chrysoeriol-6-C-glucoside | C22H22O11 | 12.28 | 463.1240 | [M+H]+ | 445, 427, 409, 397, 391, 379, 367, 343, 313 |
| **71** | Chrysoeriol-O-hexoside | C22H22O11 | 12.29 | 463.1240 | [M+H]+ | 301 |
| **72** | Diosmetin-7-rutinoside | C28H32O15 | 12.31 | 609.1820 | [M+H]+ | 463, |
| **73** | 3-Phenyl lactic acid | C19H10O3 | 12.37 | 165.0552 | [M-H]- | 147, 119, 103, 73, 59 |
| **74** | *p*-Coumaraldehyde | C9H8O2 | 12.42 | 147.0430 | [M-H]- | 119,103, 59 |
| **75** | Isoorientin-7-O-glucoside | C27H30O16 | 13.01 | 611.1570 | [M+H]+ | 449, 431, 383, 353, 329, 299 |
| **76** | Luteolin-C-[pentosyl-O-(feruoyl-O-hexoside)] | C36H36O18 | 13.02 | 757.2074 | [M+H]+ | 449, 431, 413, 309, 177 |
| **77** | Chrysoeriol-O-hexoside C-(O-feruoyl-hexoside) | C38H40O19 | 13.02 | 801.230 | [M+H]+ | 463, 445, 117 |
| **78** | Tricin-7-O-deoxyhexosyl-O-hexoside | C29H34O16 | 13.22 | 639.1925 | [M+H]+ | 493, 331 |
| **79** | Tricin-7-O-hexoside | C23H24O12 | 13.57 | 493.1330 | [M+H]+ | 331 |
| **80** | Gallic acid monohydrate | C9H16O4 | 13.71 | 187.0951 | [M-H]- | 169, 125 |
| **81** | Caffeoyl | C9H7O3 | 13.85 | 163.1123 | [M+H]+ | 145, 135, 117, 89 |
| **82** | Isovitexin-6''-O-glucoside | C27H30O15 | 14.11 | 595.1630 | [M+H]+ | 595, 433, 415, 367, 337, 313, 283 |
| **83** | Tricin-7-O-hexoside malonylated | C26H26O15 | 14.55 | 577.130 | [M+H]+ | 493, 331 |
| **84** | Luteolin-O-(O-caffeoyl-hexoside) C-hexoside | C36H36O19 | 15.58 | 773.1929 | [M+H]+ | 449, 431, 329 |
| **85** | (10E,15Z) 9,12,13-trihydroxyoctadeca-10,15-dienoic isomer I | C18H32O5 | 17.27 | 327.2171 | [M-H]- | 229, 211, 183, 171, 113 |
| **86** | Trihydroxyoctadecenoic acid | C18H34O5 | 17.93 | 329.2296 | [M-H]- | 229, 211 |
| **87** | 9-Hydroxy-12-oxo-10(E),15(Z)-octadecadienoic acid isomer I | C18H30O4 | 18.07 | 309.2078 | [M-H]- | 291, 197 |
| **88** | (10E,15Z)-9,12,13-Trihydroxy-10,15-octadecadienoic acid | C18H32O5 | 19 | 327.2015 | [M-H]- | 307, 291, 227, 213, 209, 185, 155 |
| **89** | Linolenic acid derivative isomer III | C30H38O3 | 20.05 | 445.2310 | [M-H]- | 311, 293, 277 |
| **90** | 12-Oxo-phytodienoic acid (12-OPDA) | C18H28O3 | 20.12 | 291.1910 | [M-H]- | 273, 247, 209, 165 |
| **91** | OPDA conjugate isomer II | C18H30O4 | 20.14 | 309.1913 | [M-H]- | 291, 273, 247, 209, 165 |
| **92** | *N*-Sinapoyl putrescine | C15H22N2O4 | 20.76 | 295.2245 | [M+H]+ | 207, 175, 147, 119 |
| **93** | 9-Hydroxy-12-oxo-10(E),15(Z)-octadecadienoic acid isomer II | C18H32O3 | 20.96 | 295.2256 | [M-H]- | 291, 247, 165 |
| **94** | Linolenic acid | C18H30O2 | 21.12 | 277.2149 | [M-H]- | 253, 235, 221, 197, 183, 179, 161, 113, 89 |
| **95** | Linolenic acid derivative isomer I | C33H56O14 | 21.13 | 675.3592 | [M-H]- | 415, 397, 277, 235, 89 |
| **96** | Linolenic acid derivative isomer II | C33H56O14 | 21.42 | 675.3744 | [M-H]- | 415, 397, 277, 235, 89 |
| **97** | Arachidonic acid | C20H32O2 | 21.66 | 305.2474 | [M+H]+ | 121 |
| **98** | Monogalactosylmonoacylglycerol (MGMG 18:3) | C27H46O9 | 22.28 | 559.3075 | [M-H]- | 513, 277, 253, 235 |
| **99** | Dirhamnosyl linolenic acid | C28H48O11 | 22.48 | 559.3118 | [M-H]- | 277 |
| **100** | Hydroxy octadecadienoic acid | C18H32O3 | 22.69 | 295.2273 | [M-H]- | 277, 233, 195 |
